# Regulation and function of capicua in mammals

**DOI:** 10.1038/s12276-020-0411-3

**Published:** 2020-04-01

**Authors:** Yoontae Lee

**Affiliations:** 1 Department of Life Sciences, Pohang University of Science and Technology, Pohang, Gyeongbuk Republic of Korea; 2 Yonsei University, Seoul, Republic of Korea

**Keywords:** Cancer genetics, Immunogenetics

## Abstract

Capicua (CIC) is an evolutionarily conserved transcription factor. CIC contains a high-mobility group (HMG) box that recognizes specific DNA sequences to regulate the expression of various target genes. CIC was originally identified in *Drosophila melanogaster* as a transcriptional repressor that suppresses the receptor tyrosine kinase signaling pathway. This molecule controls normal organ growth and tissue patterning as well as embryogenesis in *Drosophila*. Recent studies have also demonstrated its extensive functions in mammals. For example, CIC regulates several developmental and physiological processes, including lung development, abdominal wall closure during embryogenesis, brain development and function, neural stem cell homeostasis, T cell differentiation, and enterohepatic circulation of bile acids. CIC is also associated with the progression of various types of cancer and neurodegeneration in spinocerebellar ataxia type-1, systemic autoimmunity, and liver injury. In this review, I provide a broad overview of our current understanding of the regulation and functions of CIC in mammals and discuss future research directions.

## Gene regulation: The role of the capicua protein in mammals

A gene-regulating protein named capicua, first discovered in fruit flies, is being increasingly implicated in important roles in the early development and adult physiology of mammals. Capicua is a transcription factor, a protein that binds to specific locations in DNA and influences the rate at which genetic information is used by a cell to make RNA molecules and proteins. Yoontae Lee at Pohang University of Science and Technology in South Korea reviews current understanding of the role of capicua in mammals. Key processes that have been linked to its activity include the development of lungs, nerves, the brain and the immune system. It also influences the progression of various diseases, and has an apparent tumor-suppressing effect in cancer. Future research should focus on understanding capicua’s mechanism of action and its significance in human disease.

## Introduction

In 2000, the *capicua* (*cic*) gene was first identified in *Drosophila melanogaster* as a transcriptional repressor involved in the regulation of embryogenesis^1^. Casanova and colleagues performed a P-element screen to identify genes required for anteroposterior patterning in *Drosophila*^1^. These researchers found that a mutant embryonic phenotype characterized by a lack of abdominal segmentation but maintenance of head and tail structures was caused by a mutation in *capicua* (thus explaining the gene name, derived from the Catalan term meaning “head-and-tail”)^1^. Cic is required for organ growth and tissue patterning as well as anteroposterior and dorsoventral formation during embryogenesis in *Drosophila*^1–9^. Cic represses the expression of genes downstream of receptor tyrosine kinases (RTKs), including Torso and epidermal growth factor receptor (EGFR)^1,8^. Therefore, Cic functions as a negative regulator of the RTK signaling pathway. Moreover, RTK signaling activation promotes the degradation and/or cytoplasmic translocation of Cic via phosphorylation, thereby inducing the expression of Cic target genes downstream of RTK pathways^2,8,10^.

**Fig. 1:**
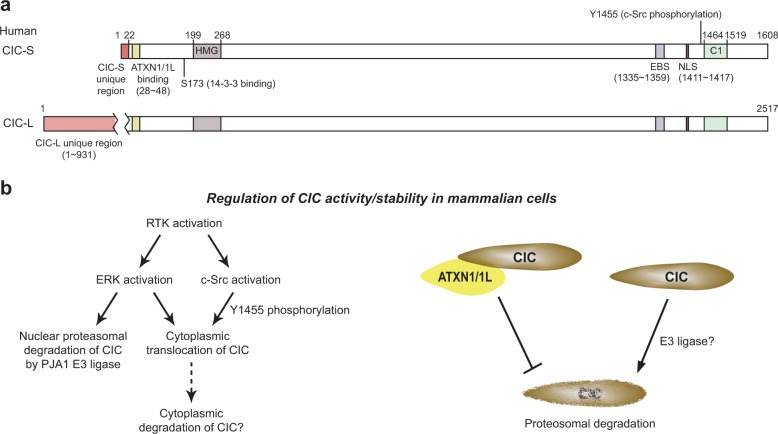
Domain features and regulation of CIC. **a** Schematic illustration of human CIC-S and CIC-L. CIC-L has a unique long N-terminal region compared with CIC-S. The amino acid regions of CIC responsible for the interaction with ATXN1/ATXN1L, 14-3-3, and ERK, the HMG box, nuclear localization signal (NLS), c-Src-mediated phosphorylation site, and C1 domain, are depicted. Numbers indicate amino acid positions. EBS: ERK binding site. **b** Regulatory mechanisms for CIC activity and stability. The left panel shows the RTK-ERK activation-mediated degradation and/or cytoplasmic translocation of CIC in mammalian cells. It is unclear whether CIC is degraded in the cytoplasm of mammalian cells. The right panel depicts the ATXN1/ATXN1L-mediated protection of mammalian CIC from proteasomal degradation. The molecular machinery mediating the degradation of CIC in the absence of ATXN1 and ATXN1L is unknown.

CIC is evolutionarily conserved from *Caenorhabditis elegans* to humans^1,11,12^. CIC exists as two isoforms, the short form (CIC-S) and the long form (CIC-L), which differ at their N-termini (Fig. 1a). CIC harbors two conserved domains, the high mobility group (HMG)-box and C1 domain (Fig. 1a), which cooperatively recognize specific octameric DNA sequences^13^. In mammals, CIC interacts with ataxin-1 (ATXN1), of which the polyglutamine (polyQ)-expanded form causes spinocerebellar ataxia type-1 (SCA1), a neurodegenerative disease^14^. CIC contributes to the pathogenesis of SCA1 in mice via interactions with mutant ATXN1^15,16^. A fusion between CIC and a transcription activator domain of double homeobox 4 (DUX4) (CIC–DUX4 fusion protein) was identified in Ewing-like sarcoma cells^17^. CIC–DUX4 fusion proteins activate the expression of *ETV1*, *ETV4*, and *ETV5*, which encode oncogenic transcription factors^18^, thereby promoting cancer progression^17^. Many studies have verified that CIC functions as a tumor suppressor in various types of cancer^19–28^. Endogenous functions of CIC have been elucidated by examinations of the phenotypes of *Cic* mutant mice. CIC deficiency results in defects in lung development, bile acid homeostasis, abdominal wall closure during embryogenesis, neuronal cell differentiation, brain development, and T cell subset differentiation^25–27,29–34^. In this review, I focus on the roles of CIC in mammals; in particular, I summarize recent studies of (1) its functions in diseases, including neurological diseases and cancer, (2) its functions in development, and (3) its underlying regulatory mechanisms in mammalian cells.

## CIC functions in diseases

### Spinocerebellar ataxia type-1 (SCA1)

SCA1 is one of nine polyQ disorders^35,36^. Expansion of the CAG repeat in *ATXN1* results in a long polyQ tract-containing mutant ATXN1, which is associated with cerebellar neurodegeneration primarily due to Purkinje cell death^35^. Phosphorylation at the S776 residue of ATXN1 is critical for the neurotoxicity of the polyQ-expanded ATXN1^37,38^. CIC binds with a high affinity to ATXN1 in human cells^14^. The CIC–ATXN1 complex is approximately 1.8 MDa in size, irrespective of the polyQ expansion in ATXN1^14^. The S776A mutation reduces the incorporation of ATXN1 into large CIC–ATXN1 complexes, implying that the interaction with CIC contributes to the neurotoxicity of the polyQ-expanded ATXN1^14^. Fryer et al. experimentally proved that CIC facilitates the pathogenesis of SCA1 using a *Cic*-deficient SCA1 mouse model (*Atxn1*^*154Q*^*; Cic-L*^*+/−*^) generated by crossing 154Q knock-in SCA1 (*Atxn1*^*154Q*^) mice with *Cic* hypomorphic (*Cic-L*^*−/−*^) mice^15^. A partial loss of CIC expression substantially attenuated the pathological and behavioral abnormalities of the *Atxn1*^*154Q*^ mice^15^. Furthermore, the expression levels of some CIC target genes were downregulated in the cerebellum of the *Atxn1*^*154Q*^ mice and were significantly rescued in the cerebellum of the *Atxn1*^*154Q*^*; Cic-L*^*+/−*^ mice^15^. These findings suggest that the polyQ-expanded ATXN1 could enhance the transcriptional repressor activity of CIC for a subset of target genes, thereby contributing to the progression of SCA1. Disruption of the interaction between the polyQ-expanded ATXN1 and CIC inhibited the SCA1 disease phenotypes in mice, suggesting that SCA1 is caused by neurotoxicity driven by a gain-of-function of the polyQ-expanded ATXN1–CIC complex^16^.

### Cancer

The first evidence for an association between CIC and cancer progression was the identification of the fusion between CIC and DUX4 as a result of a recurrent chromosomal translocation t(4;19)(q35;q13) in Ewing-like sarcomas^17^. The CIC–DUX4 chimaeras are composed of the majority of the CIC protein, except for a small portion of the C-terminus, and the C-terminal region of DUX4 involved in transcriptional activation^17^. The CIC–DUX4 fusion protein acquires transforming activity against NIH3T3 fibroblasts, indicating that *CIC–DUX4* acts as a dominant oncogene^17,39^. The chimeric proteins transcriptionally activate the expression of CIC target genes, including *PEA3* group genes that encode the oncogenic transcription factors ETV1, ETV4, and ETV5^17,18^. Several other studies have identified various additional chromosomal translocations generating *CIC–DUX4* chimeric transcripts in round cell sarcoma as well as Ewing sarcoma^40–44^. A xenograft mouse model subcutaneously injected with embryonic mesenchymal cells expressing *CIC–DUX4* developed small round cell sarcoma^45^. Another study using a xenograft mouse model orthotopically injected with NIH3T3 mouse fibroblasts expressing *CIC–DUX4* showed that the CIC*–*DUX4 proteins promote tumor growth and metastasis via the upregulation of *CCNE1* and *ETV4*, respectively, suggesting that these proteins drive tumorigenesis and metastasis in sarcomas via distinct regulatory programs^39^.

*CIC* mutations occur most frequently in oligodendroglioma. Based on high-throughput DNA sequencing analyses, *CIC* was shown to harbor point mutations in 50–70% of oligodendrogliomas carrying the codeletion of chromosomes 1p and 19q^23,24,46^. The role of *CIC* point mutations in oligodendroglioma development and progression has not been experimentally verified. However, CIC deficiency promoted gliomagenesis in a xenograft mouse model orthotopically injected with *PDGFB*-expressing neural stem cells (NSCs)^27^. The glial cell-specific deletion of *CIC* did not induce tumor formation in the mouse brain, suggesting that defects in *CIC* itself may not be sufficient to initiate oligodendroglioma^25^. Many somatic mutations in *CIC*, including truncations, insertions, and deletions, have been identified in advanced-stage human lung adenocarcinoma specimens^22^. Okimoto et al. showed that the inactivation of CIC by point mutations promotes lung cancer metastasis via derepression of ETV4, which induces the expression of MMP24^22^. The CIC-ETV4-MMP24 metastatic axis is also involved in gastric adenocarcinoma^22^. Genetic ablation of *CIC* in adult mice caused T cell acute lymphoblastic leukemia/lymphoma (T-ALL)^25,26^, suggesting that *CIC* mutations could be considered driver mutations for T-ALL in humans. T-ALL also developed in hematopoietic lineage cell-specific *Cic* null mice^26^. However, T cell-specific *Cic* null mice did not show T-ALL phenotypes up to 14 months of age^33^, suggesting that the loss of CIC in T cells may be insufficient to cause T-ALL, and CIC deficiency in other types of immune cells may contribute to disease onset in mice. Decreased CIC expression at the protein level is frequently observed in various types of cancer^19–21,28,46^. Moreover, the CIC protein levels are often not correlated with the mRNA levels within the same cancer samples, suggesting that CIC exhibits robust post-transcriptional regulation in cancer cells^19,21^. Nuclear expression of CIC decreases gradually as prostate cancer (PC) becomes more aggressive^20^. CIC levels are also substantially downregulated in hepatocellular carcinoma (HCC), glioblastoma (GBM), and colorectal cancer (CRC)^19,21,28^. The decreased expression of CIC leads to the derepression of *PEA3* group genes, thereby promoting cell growth and invasion in PC, HCC, GBM, and CRC cell lines^19–21,28^. Notably, the major *PEA3* group members (e.g., *ETV1*, *ETV4*, or *ETV5*) regulated by CIC differ among cancer cell types; the expression of *ETV5* and *ETV4* is most highly and significantly upregulated by CIC deficiency in PC and HCC cell lines, respectively^20,21^. CIC is also involved in the control of cancer stem cell properties. CIC deficiency promotes the self-renewal capacity and increases the expression of cancer stem cell markers, including EpCAM^+^/CD44^hi^/CD24^lo^ and ALDH^47,48^, via derepression of ETV4, ETV5, and SOX2 in breast cancer cell lines^49^. Consistent with this result, *CIC* levels were decreased in breast cancer patient samples with a *CD44* high and *CD24* low phenotype^49^. These data suggest that CIC suppresses breast cancer formation by restricting cancer stemness and identify CIC as a potential regulator of stem cell maintenance.

## Functions of CIC in development

### Lung development

Defective lung alveolarization has been observed in *Cic-L*^*−/−*^ mice, in which CIC-L expression is completely abolished and CIC-S expression is substantially reduced but incompletely blocked^15,31^. *Cic-L*^*−/−*^ mice exhibited perinatal lethality; approximately 83% of *Cic-L*^*−/−*^ mice died before postnatal day 14 (P14; unpublished data), and the survivors were smaller than the wild-type (WT) littermates^31^. *Cic-L*^*−/−*^ survivors had lung alveolarization defects causing air space enlargement accompanied by MMP9 overexpression in the lungs at P20^31^. Another germline *Cic* mutant (*Cic*^*△2–6/△2–6*^) mouse with deletions in *Cic* exons 2–6 (i.e., the HMG box-encoding exons), which expresses mutant CIC-L and CIC-S isoforms that lack the HMG box in the whole body, also exhibited defects in the terminal differentiation of the respiratory epithelium at embryonic day 18.5 (E18.5), potentially leading to delayed or altered alveolar maturation during postnatal development^25^. The CIC levels were relatively high in the lungs of E18.5 embryos^31^.

### Abdominal wall closure

Characterization of *Cic*^*△2–6/△2–6*^ mice revealed that CIC is required for late embryonic development. Homozygous *Cic*^*△2–6/△2–6*^ embryos were present in Mendelian ratios at E18.5 but died immediately after birth^25^. Approximately 70% of the E18.5 *Cic*^*△2–6/△2–6*^ embryos had an omphalocele, a mild type of abdominal wall closure defect^25^. In this case, the gut protrudes into the umbilical ring in the late embryonic stage. Therefore, one explanation for the early death of *Cic* mutant mice is that a part of the internal organs, such as the intestines, is cannibalized when the mother removes the placenta after birth^31,50^. The abdominal wall closure defect was also found in mice that lack the expression of ATXN1 and ATXN1-like (ATNX1L), which bind to and stabilize CIC^16,31,51^ (Fig. 1b). Approximately 45% of the E18.5 *Atxn1* and *Atxn1l* double null embryos had an omphalocele^31^. Taken together, these findings suggest that the CIC-ATXN1/ATXN1L complex is essential for normal embryogenesis and viability.

### Brain development and function

CIC is highly expressed in the brain^30,31^. This molecule has been implicated in granule cell development based on the observation that *Cic* is highly expressed in immature granule cells in the cerebellum, hippocampus, and olfactory bulb^12^. A study of *Cic* mutant mice uncovered a critical role of CIC in brain development and function^32^. The deletion of *Cic* in the forebrain significantly reduced the thickness of cortical layers 2–4 and the dentate gyrus, potentially due to defects in the maintenance of postmitotic neurons^32^. The layer 2/3 pyramidal neurons of the forebrain-specific *Cic* null (*Cic*^*f/f*^*;Emx1-Cre*) mice also had defective dendritic branching^32^. CIC deficiency in the forebrain caused learning and memory deficits, and a loss of CIC in the hypothalamus and medial amygdala led to defects in social interactions^32^. Consistent with these mouse data, *de novo* heterozygous truncating mutations in *CIC* are associated with autism spectrum disorder, developmental delay/intellectual disability, seizures, and attention deficit hyperactivity disorder in humans^32^.

CIC is also associated with NSC maintenance and differentiation. *Cic* null NSCs presented EGF-independent hyperproliferative characteristics^27^. Hyperproliferation of NSCs by the loss of *Cic* was also confirmed in E13 embryos by a 5-ethynyl-2′-deoxyuridine (EdU) labeling experiment^29^. Upon the induction of differentiation in vitro, *Cic* null NSCs could not differentiate into mature oligodendrocytes and instead were maintained in an oligodendrocyte progenitor cell (OPC)-like stemness state^27^. A similar result was obtained using another forebrain-specific *Cic* null (*Cic*^*f/f*^*;Foxg1-Cre*) mouse model, in which Olig2^+^Sox2^+^ cells and Olig2^+^Pdgfra^+^ OPCs are increased and CNPase^+^ immature oligodendrocytes are decreased in the cortex^29^. Moreover, CIC deficiency enhanced the self-renewal capacity and promoted the symmetric division of NSCs^29^. Mechanistically, the derepression of *Etv5* mediated the effects of CIC deficiency in NSCs^29^. Thus, CIC is a key transcription factor that controls brain development and function as well as the pathogenesis of neurological disorders.

### Immune cell development and function

Park et al. investigated the role of CIC in the immune system by generating and characterizing hematopoietic lineage cell-specific *Cic* null (*Cic*^*f/f*^*;Vav1-Cre*) mice^33^. These mice had lymphoproliferative disorder-like symptoms at 9 weeks of age, as evidenced by an increased splenocyte count mainly due to the expansion of the B220^+^ B cell population and hyperglobulinemia. *Cic*^*f/f*^*;Vav1-Cre* mice eventually developed systemic autoimmune-like phenotypes, including the enlargement of secondary lymphoid organs; increased anti-dsDNA antibody serum levels; immune cell infiltration into various organs, including the liver, lung, and kidney; and IgG deposition at the glomeruli of the kidney. T cell-specific *Cic* null (*Cic*^*f/f*^*;Cd4-Cre*) mice also exhibited similar phenotypes to *Cic*^*f/f*^*;Vav1-Cre* mice, suggesting that CIC deficiency in T cells is critical for the induction of autoimmune-like symptoms^33^. CIC deficiency promotes the differentiation of follicular helper T (Tfh) cells^33^, which play a pivotal role in the germinal center reaction to produce isotype class switched high affinity antibodies against specific antigens^52^. At the molecular level, *Etv5* is a critical target gene of CIC for the regulation of Tfh cell differentiation^33^. ETV5 levels were significantly upregulated in *Cic* null Tfh cells compared with WT cells. Adoptive transfer experiments using OT-II cells, ovalbumin-specific T cell receptor-expressing CD4^+^ T cells, revealed that ETV5 overexpression promotes Tfh cell development and that the knockdown of ETV5 substantially rescues the enhanced Tfh cell differentiation of *Cic* null OT-II cells^33^. These results indicate that the CIC-ETV5 axis controls Tfh cell development. Park et al. also proposed that *Maf*, which encodes a transcription factor that promotes Tfh cell differentiation^53^, is a target of ETV5 in CD4^+^ T cells under STAT3 activation^33^.

CIC is also involved in maintaining homeostasis of bone marrow hematopoietic stem and progenitor cells (HSPCs) and early T cell development^26^. Analyses of bone marrow and thymic cells in adult stage-specific (*Cic*^*f/f*^*;UBC-Cre/ERT2*) and endothelial and hematopoietic lineage cell-specific (*Cic*^*f/f*^*;Tek-Cre*) *Cic* null mice have shown that the number of HSPCs, including hematopoietic stem cells (HSCs) and multipotent progenitors (MPPs), is reduced, whereas the frequency of thymic double negative 1 (DN1) cells is significantly increased^26^. The frequency of early T cell precursors (ETPs), a subset of DN1 cells from the bone marrow that remain pluripotent, is also elevated in the thymus of *Cic*^*f/f*^*;UBC-Cre/ERT2* mice, suggesting that CIC regulates the self-renewal capacity of stem-like cells^26^.

CIC has been implicated in the development of CD8^+^ resident memory T (Trm) cells in the liver^34^. *Cic-L*^*−/−*^ mice exhibit liver damage, as evidenced by increases in serum alanine transaminase (ALT) and hepatic proinflammatory cytokine expression levels^30^. These mice also have defects in the enterohepatic circulation of bile acids accompanied by the downregulation of several key genes involved in bile acid biosynthesis and transport in the liver^30^. These liver dysfunctions are not due to a CIC deficiency in hepatocytes because liver-specific *Cic* null (*Cic*^*f/f*^*;Alb-Cre*) mice do not recapitulate these phenotypes^34^. *Cic*^*f/f*^*;Cd4-Cre* mice have increased serum ALT and hepatic proinflammatory cytokine expression levels, indicating that CIC-deficient T cells cause inflammatory liver injury^34^. CIC deficiency promotes the formation of liver CD8^+^ Trm-like cells expressing surface markers, such as CD69^+^, CD49a^+^, CXCR6^+^, CXCR3^+^, and CD103^-^, in a cell intrinsic manner^34^. Moreover, the suppression of liver CD8^+^ Trm-like cell formation dramatically mitigated liver injury phenotypes in *Cic*^*f/f*^*;Cd4-Cre* mice treated with acetaminophen, which induces acute liver injury, suggesting that the increased CD8^+^ Trm-like cell population in the liver is responsible for the CIC deficiency-induced liver injury^34^. Mechanistically, the CIC–ETV5 axis controls liver CD8^+^ Trm-like cell differentiation. The derepression of ETV5 induces the expression of HOBIT, a transcription factor required for Trm cell development^54^, in *Cic* null CD8^+^ T cells, thereby promoting Trm cell differentiation^34^.

## Regulation of CIC

RTK-RAS-MAPK pathways suppress CIC activity via the cytoplasmic translocation and/or degradation of CIC (Fig. 1b). This regulatory mechanism was originally discovered in studies of CIC expression patterns in *Drosophila* embryos. Torso RTK signaling in the early embryo leads to the degradation of CIC, whereas EGFR signaling in the ovarian follicle induces the partial relocalization of CIC to the cytoplasm^10,55^. EGF treatment resulted in the phosphorylation of human CIC-S at 20 different serine/threonine residues, presumably by ERK and p90^RSK^, a kinase activated by ERK^56^. In particular, p90^RSK^-mediated phosphorylation of S173 is critical for 14–3–3 binding (Fig. 1a), which inhibits CIC binding to target DNA sequences^56^. S1409 phosphorylation prevents the binding of importin α4/KPNA3 to the nuclear localization signal of CIC^56^. However, the disruption of the CIC-KPNA3 interaction does not affect the nuclear localization of CIC-S^56^, suggesting that other transport-related factors might be required for the cytoplasmic translocation of CIC in mammalian cells. ERK binds to the C-terminal region of human CIC-S containing residues 1335–1359 (prior to the C1 domain; Fig. 1a)^57^. EGFR stimulation decreased CIC levels in mammalian cells^15,19,22^. The inhibition of ERK by treatment with MEK1/2 inhibitors increased the levels of nuclear CIC-S at the expense of cytoplasmic CIC expression in pancreatic cancer cells^58^, suggesting that ERK regulates the subcellular localization of CIC (Fig. 1b). Moreover, EGFR-activated c-Src tyrosine kinase mediates cytoplasmic translocation of CIC-S via phosphorylation of the Y1455 residue^59^ (Fig. 1a, b). CIC is degraded in the nucleus upon EGFR-ERK activation^19^. In this process, the nuclear E3 ligase PRAJA1 (PJA1) polyubiquitylates CIC, leading to the proteasomal degradation of CIC in the nucleus^19^ (Fig. 1b). DNA binding of CIC is a prerequisite for the PJA1-mediated polyubiquitylation of CIC^19^. In addition, PJA1 recognizes the S173 residue of CIC-S to interact with CIC^19^. Since 14-3-3 also binds to S173-phosphorylated CIC-S to control the transcriptional repressor activity of CIC^56^, crosstalk between 14-3-3 and PJA1 might be involved in the regulation of CIC activity and/or stability.

Another regulatory mechanism underlying CIC activity is the ATXN1/ATXN1L interaction-mediated stabilization of CIC (Fig. 1b). Both ATXN1 and its homolog ATXN1L interact with and stabilize CIC^31^. The AXH domain of ATXN1/ATXN1L and the highly conserved N-terminal region of CIC-S, including amino acid residues 28–48, mediate their interaction^60^ (Fig. 1a). ATXN1L plays a more pivotal role in the stabilization of CIC than ATXN1; CIC levels decreased more substantially in response to the loss of ATXN1L than to the loss of ATXN1, leading to substantial derepression of CIC target gene expression^31,61^. In the absence of ATXN1L, CIC becomes unstable, resulting in proteasomal degradation^61^. ATXN1L also promotes CIC binding to the target gene promoter regions^61^. However, the reason for the relative importance of ATXN1L for CIC stabilization and function is unclear.

Long noncoding RNA (lncRNA)-mediated regulation of *CIC* expression has been reported^62^. The levels of *CIC* and lncRNA-*AC006129.1*, of which genomic locus is close to *CIC* in chromosome 19, were significantly decreased and increased in samples from schizophrenia patients, respectively^62^. *AC006129.1* transgenic mice exhibited social interaction deficits, spatial working memory impairments, and sensorimotor gating disruption accompanied by upregulation of inflammatory response genes, including *SOCS3* and *CASP1*, which are CIC target genes^62^. The overexpression of *AC006129.1* downregulated CIC levels in both mouse and human cells^62^, suggesting that this lncRNA-mediated transcriptional repression of CIC expression might be conserved in mammals. Mechanistically, *AC006129.1* recruits DNA methyltransferases 1 and 3a (DNMT1 and DNMT3a) and induces DNA methylation of *CIC* promoter regions^62^. The *AC006129.1*-mediated suppression of CIC expression leads to derepression of *SOCS3* and *CASP1*, potentially contributing to the pathogenesis of schizophrenia^62^.

## Concluding remarks

CIC has multiple roles in various developmental processes and in the pathogenesis of various diseases. CIC is believed to function as a tumor suppressor in various types of cancer and is a regulator of embryogenesis, brain and immune cell development, and stem cell maintenance. Our current understanding of CIC functions in mammals is largely limited to processes regulated by the CIC-ETV1/ETV4/ETV5 axis. Many molecular studies of mammalian cells have identified additional target genes of CIC, such as *Spry4*, *Dusp4*, *Dusp6*, *Spred1*, *Ccnd1*, *Ccne1*, and *Per2*^15,17,27,39,63,64^. It will be important to clarify the effects of CIC regulation of various target genes at both the cellular and organismal levels. Furthermore, the mechanism by which CIC regulates target gene expression remains largely unclear and should be a focus of future research. CIC was shown to recruit the histone deacetylase complex to repress the expression of target genes in stem cells^64^. Another study proposed that CIC has dual functions as a transcriptional activator as well as a repressor^27^. There are several unanswered questions regarding the regulation of CIC activity. For example, which factors mediate the cytoplasmic translocation of CIC upon the activation of RTK signaling? How does ATXN1L stabilize CIC at the molecular level? Which transcription factors control the expression of *CIC*? These unresolved issues need to be addressed for a comprehensive understanding of the CIC-mediated regulation of biological processes. Finally, CIC is emerging as a key determinant of immune responses. A few studies have recently uncovered the roles of CIC in the development of T cell subsets^26,33,34^. However, the function of CIC in other types of immune cells, including B cells, dendritic cells, and macrophages, has not been established. Comprehensive studies of CIC functions in various types of immune cells will improve our understanding of the pathogenesis of immune disorders, such as autoimmune diseases and lymphomas, at the molecular level.
